# SiMON-PRO-Hematology®: Digital techology for pharmacotherapeutic and patient reported outcomes monitoring in patients with hematologic malignancies

**DOI:** 10.1016/j.rcsop.2026.100819

**Published:** 2026-06-26

**Authors:** Alejandro Martínez Pradeda, Elena Fernández Gabriel, Sergio Jimeno Aguado, Ana Sanclaudio Luhia, Víctor Noriega Concepción, Luis Margusino-Framiñán

**Affiliations:** a Pharmacy Service, Complexo Hospitalario Universitario de A Coruña, Universidade de A Coruña, Research Group of Hospital Pharmacy, Biomedical Research Institute A Coruña (INIBIC), A Coruña University Hospital (CHUAC), Sergas, A Coruña, Spain; b Pharmacy Service, Complexo Hospitalario Universitario de A Coruña, A Coruña, Spain; c Information Systems Unit, Complexo Hospitalario Universitario de A Coruña, A Coruña, Spain; d Hematology and Hemostasia Service, Complexo Hospitalario Universitario de A Coruña, Universidade da Coruña (UDC), Instituto de Investigación Biomédica de A Coruña, A Coruña, Spain

**Keywords:** Hematologic malignancies, Patient-reported outcomes, Digital technoloty, Pharmaceutical care, Electronic health records, Real-time monitoring

## Abstract

**Introduction:**

Advances in hematologic malignancy treatment have improved survival, but patients often experience treatment-related toxicities. Traditional clinical follow-up often overlooks the patient's subjective experience, highlighting the need for integrated patient-reported outcome measures (PROMs). The primary objective of this study was to describe the design and implementation of the SiMON-PRO-Hematology® system, a comprehensive information tool supporting the pharmacotherapeutic follow-up of patients with hematological malignancies. A secondary objective was to detail the process for PROM collection

**Methods:**

SiMON-PRO-Hematology was designed by a multidisciplinary team of pharmacists, hematologists, nurses, and software engineers. This group defined the functional requirements of the system, as well as the demographic, clinical, laboratory, and pharmacotherapeutic variables and disease-specific PROMs relevant to hematologic malignancies. The system was structured into three main areas: Clinical care (assistance), management, and research. No primary data were collected; therefore, ethical approval was not required

**Results:**

SiMON-PRO-Hematology automates PROM collection via tablet devices completed independently by patients. The platform integrates PROMs with baseline clinical data and laboratory results, generates real-time alerts, and supports structured pharmaceutical interventions. Business intelligence tools enable analysis of patient evolution, treatment adherence, and cohort comparisons. Implementation allows standardized, longitudinal follow-up, enhancing detection of safety and effectiveness signals while streamlining workflows and minimizing documentation duplication. Unlike existing tools, SiMON-PRO-Hematology integrates independently reported PROMs within a broader, adaptable monitoring system tailored to diverse hematologic malignancies

**Discussion:**

This approach ilustrates how a fully integrated digital platform is designed to potentially enhance the quality and efficiency of pharmaceutical care, providing a technical framework to support proactive interventions, longitudinal monitoring, and patient-centered outcomes

## Highlights

• A pharmacist-led digital system was developed for PROM-based follow-up in hematologic care.
• The platform integrates PROMs, clinical and laboratory data into routine pharmacy practice.
• Automated PROM assignment and alerts support proactive pharmacotherapeutic interventions.
• The system enables standardized, longitudinal monitoring while reducing documentation burden.
• The model supports patient-centered care and real-world data generation in hospital pharmacy.

## 1 Introduction

Advances in hematologic malignancy treatment have transformed the clinical landscape, with some leukemias and lymphomas now considered chronic diseases.^1^

This shift brings challenges, as patients face treatment-related toxicities, physical sequelae, and emotional burdens. Long-term therapies, though often less toxic than intensive chemotherapy, can still impair quality of life (such as imatinib-related muscle pain or ibrutinib-related arthralgia).^2^, ^3^, ^4^, ^5^, ^6^

The growing range of treatments—including tyrosine kinase inhibitors, monoclonal antibodies, and CAR-T cells—offers improved disease control but also introduces diverse adverse effects requiring careful monitoring. Patient management thus extends beyond achieving remission to include toxicity and long-term quality-of-life considerations. When multiple strategies exist (such as finite combined treatment vs. monotherapy until progression), QoL is crucial for comparative decision-making. Tools that assess not only efficacy but also the subjective impact of therapy are increasingly important. Patient-reported outcome measures (PROMs) capture symptoms, well-being, and side effects over time, supporting closer monitoring and informed clinical decisions. International guidelines now recommend PROMs in both solid and hematologic malignancies.^7^, ^8^

With the development of new PROMs questionnaires and the growing recognition of their clinical value,^8^ their application has expanded significantly. In clinical trials, the value of PROMs in treatment monitoring and symptom management is well established^9^, ^10^, ^11^ However, several studies have shown that the collection of PROMs is not yet a recognizable reality for patients with hematologic malignancies in routine clinical practice.,^12^ Multiple barriers have been identified, primarily those related to healthcare system constraints,^13^, ^14^, ^15^, ^16^ as well as social and patient-related factors.^17^

Nevertheless, some authors have reported positive real-world experiences that demonstrated favorable outcomes,^12^,^18^). Furthermore, recommendations have been published to support their integration into daily clinical practice, emphasizing that this can be achieved without drastically altering existing workflows.^19^, ^20^

Several studies have described available tools for collecting PROMs,^21^, ^22^, ^23^ but these tools are usually developed by external entities, which introduces complexities in terms of integration with patients' electronic health records (EHR), as well as legal issues that may limit their accessibility. In addition, the implementation of these systems often entails a financial cost that not all healthcare organizations can afford, especially in settings with limited resources or where digital infrastructure is underdeveloped.

A previous initiative focused on hepatitis C led to the development of the SiMON® (acronym for “Sistema Inteligente de MONitorización de pacientes”, in English “intelligent system for patient monitoring”), designed to integrate comprehensive patient clinical data and improve pharmacotherapeutic care.^24^ While originally designed for viral hepatitis, the platform has since evolved into a versatile system adapted for other high-complexity areas, such as hematologic malignancies, incorporating patient-reported outcomes (PROMs) into routine pharmaceutical care.

This study primarily aims to describe the design and implementation of the SiMON-PRO-Hematology® system, a comprehensive information tool aimed at supporting the pharmacotherapeutic follow-up of patients with hematological malignancies. As a secondary objective, this study seeks to detail the process or circuit for the collection of these PROMs.

## 2 Methods

### 2.1 Creation of a multidisciplinary working group

A multidisciplinary working group, including pharmacists, hematologists, nurses, and software engineers, led by the pharmacist in charge of the hematology pharmacy unit, was established to design the SiMON-PRO-Hematology system using the previously described SiMON-VC tool^24^ as a framework. The team defined functional requirements, selected relevant demographic, clinical, and pharmaceutical variables, chose PROMs questionnaires specific to hematologic diseases, and developed a systematic procedure for their collection via telepharmacy. Usability and functionality were validated through pilot testing with real patients. Some variables are detailed in Annex I using the Chronic Lymphocytic Leukemia dataset as an example. The design process was completed in one year.

### 2.2 Information system requirements

The system's functional requirements were grouped into three main areas, which in turn corresponded to the program's tabs, for intuitive navigation and usability: Assistance, Management, and Research requirements. The requirements for each category are as follows:

1. ASSISTANCE:

• Registration of hematologic cancer patients undergoing treatment, with the automatic integration of baseline and epidemiological data from their EHR.
• Documentation of follow-up visits in the pharmaceutical care consultation, automatically incorporating hematologic, biochemical, and microbiological test results from laboratory databases.
• Completion of PROMs questionnaires on an independent electronic device accessible to the patient, with direct upload of results into the system, without increasing consultation time.
• Automatic assignment of pre-selected PROMs according to each patient's diagnosis, ensuring that the appropriate questionnaires are presented at each visit based on the follow-up schedule.
• Automated reminders to review and follow up on PROMs and PREMs (Patient-Reported Experience Measures) according to the periodicity established by the healthcare team.
• Generation of automatic clinical alerts when PROMs results indicate the need for medical or pharmaceutical intervention.
• Generation of automatic pharmaceutical events and alerts related to treatment efficacy and safety.
• Estimation of patient adherence to oncologic treatment.
• Documentation of visits to the hospital pharmacy within the EHR by creating an automated summary of clinical follow-up, based on all collected information (PROMs results, laboratory data, etc.), allowing seamless integration into the patient's EHR without duplication.
  2. MANAGEMENT:
• Generation of an Activity Dashboard and a Quality Control Dashboard, including automatic generation of indicators and alerts based on established standards.
  3. RESEARCH:
• Statistical analysis of data using Business Intelligence (BI) tools.
• Maximum versatility in data usage.

As no primary data will be collected for the purposes of this study, no formal ethical approval is required.

## 3 Results

### 3.1 Structure of SiMON-PRO-hematology

**Fig. 1:**
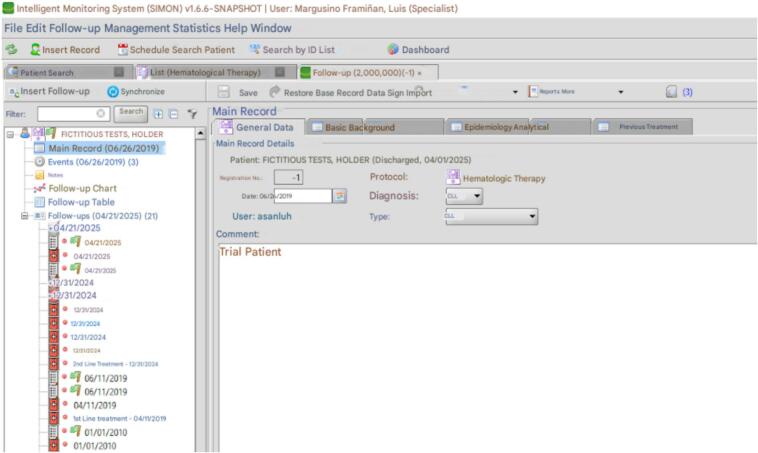
Screenshot of the SiMON-PRO-Hematology main menu. The original software interface is in Spanish; the image shown corresponds to an English-translated version for publication purposes. The upper section shows the three functional modules: the assistance module (Register Patient, Schedule, Search Patients), the management module (Dashboard), and the research module (Import). The lower section displays the assistance module, with a data tree on the left organizing patient data into a Main Record, Events, Notes, and Follow-ups. The main panel presents the Main Record, with tabs for General Data, Baseline, and Clinical information.
• **Main Record:** A record is created for each hematologic malignancy, automatically activating condition-specific baseline data tabs when the diagnosis is selected.
• **Events:** The system automatically generates alerts and events related to treatment efficacy and safety, which have been previously defined. This field collects all the events that are recorded in a single patient's record, including those entered manually by the pharmacist and those automatically extracted from the EHR or other sources.
• **Notes:** Free-text, non-coded field.
• **Follow-up Chart:** Visualizes changes in measurable parameters over time in graphs.
• **Follow-up tables:** Numerical entries used to generate monitoring graphs.
• **Follow-ups**: Lists all consultations in the Hospital Pharmacy Unit. Each entry corresponds to a pharmaceutical care consultation —whether in-person or by phone—or to an administration of parenteral treatments. These entries are organized by treatment line, providing a structured record of the patient's care evolution.

The SiMON-PRO-Hematology program features a main menu accessible through individualized credentials. Access to different protocols is tailored to the user profile. The structure of the main menu of SiMON-PRO-Hematology allows for a clear distinction between the three functional areas of the program, as defined during the design phase. Each option corresponds to a specific module:

• **Assistance Module:** Includes the **Register Patient**, **Schedule**, and **Search Patients** options, which focus on direct patient management and follow-up.
• **Management Module:** Corresponds to the **Dashboard** option, designed for monitoring activity and quality indicators.
• **Research Module:** Facilitates access to clinical data through the “Import” function and enables advanced analysis using BI tools. These tools allow identification of patient evolution patterns, generation of detailed treatment reports, evaluation of long-term adherence, comparisons between cohorts, and the creation of dashboards and graphs to visualize patient status and disease progression.
  Upon registering a new patient or selecting an existing one, the assistance module is accessed, organized as a data tree (Fig. 1, lower section):

### 3.2 PROMs integration and automatic assignment

The SiMON-PRO-Hematology program integrates the collection of PROMs questionnaires tailored to the patient's pathology. These questionnaires are pre-selected, and the system automatically assigns the forms to be completed at each visit. Assignment is based on the schedule established by the interdisciplinary team, ensuring that quality-of-life and symptom data are collected at appropriate times.

### 3.3 Reminders for healthcare professionals

SiMON-Hematology generates automatic reminders for pharmacists and other healthcare professionals involved in clinical follow-up. These reminders ensure that PROMs questionnaires are reviewed and that the entered data are analyzed for potential interventions. Reminders also include alerts about delays in PROMs collection, ensuring timely assessment of the patient's condition.

### 3.4 Clinical alert generation

One of the system's main functions is the automatic generation of clinical alerts. When PROMs results or clinical data indicate the need for intervention (eg, symptom worsening, severe toxicities, or poor treatment adherence), the system sends alerts directly to the professionals responsible for the case. This enables a rapid and timely clinical response, improving patient management and reducing potential complications.

### 3.5 Automated clinical summary

In addition to managing data collection and alert generation, SiMON-Hematology allows for the creation of an automated clinical summary. This summary includes all data recorded during the patient's follow-up, including PROMs results, laboratory tests, and clinical evaluations. The summary can be directly integrated into the patient's EHR, avoiding duplication and ensuring consistency across systems.

### 3.6 Features of SiMON-hematology

#### 3.6.1 Monitoring and follow-up process for hematological patients using SiMON-PRO-hematology

The implementation of the SiMON-PRO-Hematology system has established a pharmaceutical care circuit focused on the comprehensive monitoring and follow-up of patients with hematologic malignancies. This process begins at the consultation and is fully documented on the platform, facilitating efficient and proactive management.

**Fig. 2:**
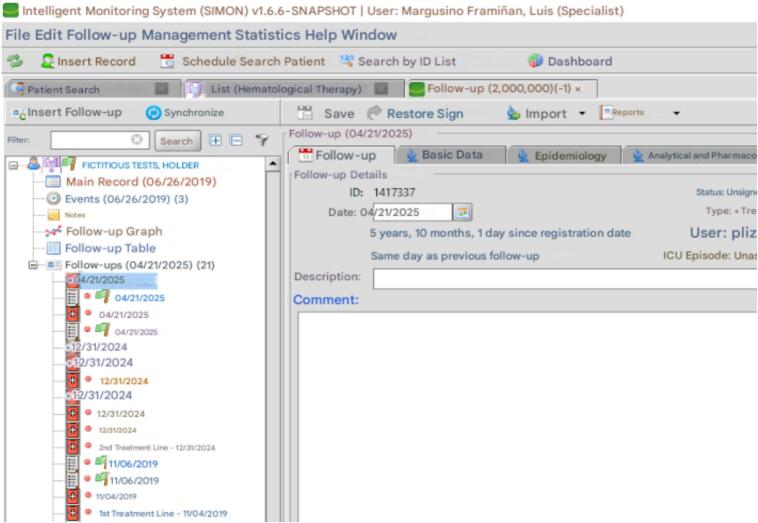
A view of a “Follow-up” in the SiMON-PRO-Hematology system. The original software interface is in Spanish; the image shown corresponds to an English-translated version for publication purposes. The interface shows the tabs that guide the pharmacist through the care process, allowing for the recording of basic data, the patients evolution, and the management of clinical events.

When a patient is seen, the pharmacist enters a new “Follow-up,” which can be one of two types: in-person or by phone. Within each follow-up, several tabs are accessed in a logical order that reflects the consultation process (Fig. 2).

**Fig. 3:**
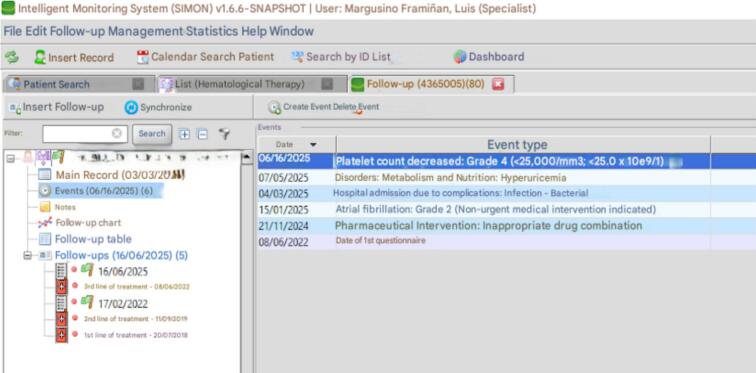
The events section of the patient's profile. The original software interface is in Spanish; the image shown corresponds to an English-translated version for publication purposes. This view compiles all the alerts and events recorded for the patient (both automatic and manual), offering a complete history of safety and efficacy interventions.
• Questionnaire Tab: The results of the PROMs and PREMs questionnaires are automatically integrated into this tab when the patient completes them using assigned tablet devices (Fig. 3, Fig. 4). This ensures that if any result is anomalous or notable, an alert is generated.

• Basic Data Tab: Here, essential data is recorded, such as medication dosage, the presence of “subjective” adverse effects, the initiation of new treatments that may cause drug interactions, and the patient's evolution based on imaging tests. The system also calculates and records treatment adherence. The entry of this basic data automatically generates efficacy, safety, or pharmaceutical care events (such as partial response, poor adherence, vomiting, dose reduction, etc.).
• Laboratory Data Tab: This section allows for the automatic import of the patient's corresponding laboratoy results. Upon importing the results, the program generates automatic alerts based on a pre-formatted database, which includes all adverse events from the CTCAE (Common Terminology Criteria for Adverse Events^25^) with their respective grades (Fig. 3).

**Fig. 4:**
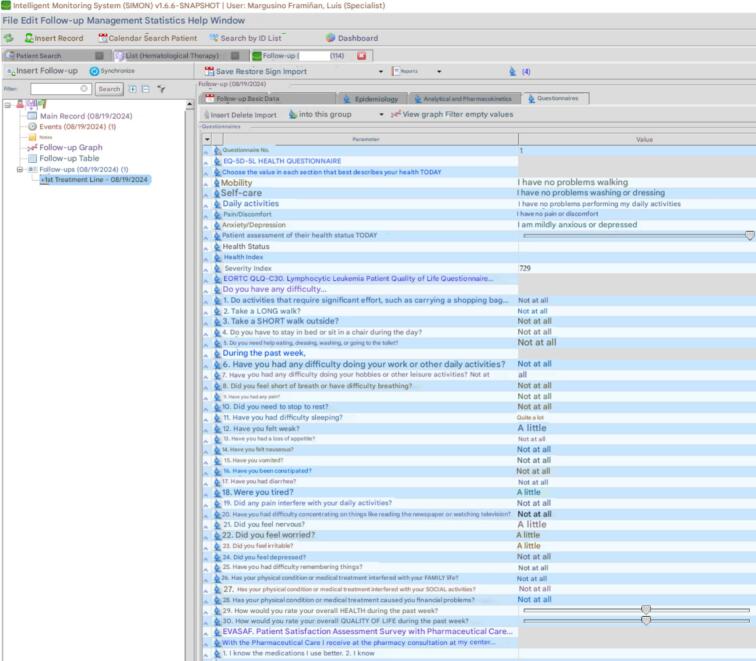
The questionnaire tab in SiMON-PRO-Hematology. The original software interface is in Spanish; the image shown corresponds to an English-translated version for publication purposes. Questionnaire items correspond to literal translations of the original Spanish versions. It displays the results of the PROMs and PREMs questionnaires completed by the patient, with automatic alerts that are activated if the results are anomalous or require attention.

The system also captures other automatic events directly from the patient's EHR, such as hospital admissions or delays in treatment administration (Fig. 3). All these automatically generated alerts and events allow for additional clarification through an observations field, which facilitates the pharmacist's work.

Upon completing the follow-up, the program generates a summary document with all the entered fields, signed and dated by the pharmacist conducting the follow-up, which is automatically uploaded to the patient's EHR to minimize duplication of records.

#### 3.6.2 Development of a reporting framework for PROMs and PREMs

The main barriers to systematic PROMs and PREM collection are high resource demands (time and staff) and the complexity of adapting questionnaires to individual visits. To address these challenges, the multidisciplinary working group performed a contextual analysis and designed an optimization strategy, extending SiMON-PRO-Hematology to incorporate QoL assessments.

The system uses tablets provided by the healthcare center for patient use during visits. These devices integrate with the hospital's appointment scheduling and EHR systems, allowing patient identification via registration number or automatically generated QR code.

**Fig. 5:**
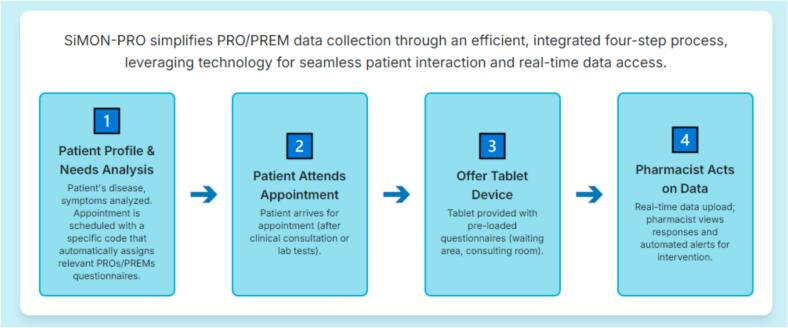
Process for selecting and collecting PROM and PREM data. This diagram illustrates the efficient and integrated workflow of the SiMON-PRO-Hematology system for the collection of Patient-Reported Outcomes Measures (PROMs) and Patient-Reported Experience Measures (PREMs). This four-step process is designed to optimize patient interaction and ensure real-time access to crucial information for pharmacotherapeutic follow-up. It enables the automatic assignment of specific questionnaires to each patient based on their profile, autonomous data collection via tablets, and immediate visualization of responses and alerts by the pharmacist, facilitating proactive and personalized clinical decision-making. Tablets are configured with restricted access, allowing only the use of the questionnaire and medical record systems, with no access to external applications or web browsing. All devices are remotely monitored to ensure proper functionality and to prevent potential security breaches.The questionnaires are presented in a user-friendly format, employing scales and closed-ended questions to facilitate understanding and efficient completion. Once completed, the data are automatically and securely transferred to the EHR without requiring manual input from healthcare professionals.

A set of clinical profiles was defined, each linked to specific pathologies and patient types. Each profile is automatically assigned a predefined set of PROMs and PREM questionnaires when a patient is scheduled, eliminating manual selection. Patients can complete the questionnaires on tablets during the visit, in the waiting area, or auxiliary rooms, depending on workflow (Fig. 5).

The system incorporates advanced cybersecurity measures, including multifactor authentication, end-to-end encryption, and restricted tablet access, preventing use of external applications (detailed technical specifications of this infrastructure are provided in the **Supplementary Material II**). Devices are remotely monitored for proper functionality. Questionnaires use user-friendly scales and closed-ended questions, and completed data are securely and automatically transferred to the EHR.

#### 3.6.3 Dashboard / control panel for activity, quality, and safety

The SiMON-PRO-Hematology program includes an advanced functionality that enables the automated and flexible creation of indicator dashboards related to healthcare activity, process quality, and patient safety. This tool not only facilitates the calculation of key metrics and the detection of deviations from established thresholds, but also reinforces the role of SiMON-PRO-Hematology as a decision support system.

In the specific context of PROMs and PREMs follow-up, this feature is especially useful, as it enables early detection of delays in the collection of protocolized questionnaires—essential for conducting homogeneous comparisons and outcome analyses.

Additionally, the system includes an internal verification mechanism that monitors the integrity and consistency of the baseline data recorded, allowing immediate identification of patients with incomplete or incorrectly entered information.

### 3.7 Research

One of the main strengths of this information system is its ability to harness the large volume of registered and coded data—whether manually entered or automatically generated in SiMON-PRO-Hematology—for research purposes. This functionality enables the evaluation of health outcomes related to medication use and/or pharmaceutical care provided, using Big Data and BI tools.

Moreover, the platform allows the export of data into formats compatible with major statistical analysis programs, such as Excel® or SPSS®.

## 4 Discussion

The implementation of SiMON-PRO-Hematology represents a major step in the digital transformation of pharmaceutical care for patients with hematologic malignancies. The platform enables comprehensive, standardized, longitudinal follow-up by consolidating structured clinical data, laboratory values, and Patient-Reported Outcomes (PROMs) in a single system.

It supports early detection of safety and efficacy signals, monitors adherence, and identifies toxicities and QoL deterioration. EHR integration delivers near-real-time alerts for unexpected events, minimizing duplication and enhancing documentation. Embedded BI tools facilitate data analysis, trend identification, quality assessment, and Real-World Evidence studies, while structured reporting of interventions and CTCAE-based adverse events highlights the pharmacist's role.^26^, ^27^

Beyond clinical monitoring, SiMON-PRO empowers patients by involving them directly in disease management. Coupled with prior education, this enhances patient understanding of their condition and side effects, improving accurate reporting and supporting shared decision-making and treatment adherence.

Reporting PROMs has been shown to improve symptom identification and doctor–patient communication.^28^, ^29^, ^30^ In recent years, many digital tools have been developed to collect PROMs, most of which focus on patient-entered data completed independently, often at home. However, unlike these standalone systems, SiMON-PRO-Hematology goes beyond simple data collection, providing a comprehensive platform for pharmacist-led pharmacotherapeutic follow-up.. It integrates PROMs and PREMs as just one facet of a broader patient overview, enabling the pharmacist to manage a complete spectrum of clinical information, rather than just isolated QoL data. Secondly, EHR-integrated PROMs systems have shown success in cancer symptom management across varied healthcare settings,^31^, ^32^ SiMON-PRO-Hematology is specifically developed and tailored for patients with hematologic malignancies. This specialized focus allows for the capture and interpretation of highly specific and critical data points essential for this population. For example, it integrates specific disease markers, monitors drug plasma concentrations, and automatically grades laboratory toxicities according to CTCAE criteria.^25^ Furthermore, it incorporates disease-specific QoL questionnaires, such as the EORTC QLQ-C30 paired with its hematology-specific modules (such as, EORTCQLQ-MY20 for multiple myeloma), which provide nuanced insights beyond general oncology PROMs. This bespoke design ensures that the data collected is not only comprehensive but also directly actionable and highly relevant to the unique challenges of hematological care.

Despite its numerous benefits, the implementation of SiMON-PRO-Hematology presents certain limitations. In the first place, it is important to clarify that while the system automates and standardizes all objectively measurable and electronically available data (such as laboratory parameters, adherence calculations, and automatic CTCAE grading), it still relies on manual entry for specific non-parameterized clinical variables. This manual documentation is reserved exclusively for subjective toxicities, physical examination findings, or clinical impressions that inherently require professional assessment and grading by the pharmacist (e.g., nuanced clinical interpretation of symptoms like fatigue or diarrhea). Rather than a gap in automation, this design preserves essential clinical judgment where automated metrics are insufficient or not possible. Likewise, dependence on software engineer support for structural or functional modifications limits the autonomy of clinical users to make agile changes. Another limitation regarding telepharmacy integration is the current restriction on collecting PROMs and PREMs remotely, which is limited by institutional cybersecurity and firewall frameworks Although an advanced version of the platform capable of supporting secure remote data collection has already been developed and in the future, new telepharmacy procedures will be developed to remotely integrate protocols with the required cybersecurity conditions Furthermore, the generalizability of the system may be limited in settings without integrated digital infrastructure or EHR. Its reliance on predefined questionnaire circuits, while facilitating structured monitoring, restricts flexibility for individualized symptom assessment. Other factors that can negatively impact implementation include the need for training and familiarization for healthcare staff, which poses a barrier in settings with high turnover t A limitation of this study is that the collection of PROMs and PREMs through a telemedicine platform required participants to possess sufficient digital literacy, health literacy, and cognitive capacity to understand and complete the questionnaires. Therefore, patients with limited technological skills, low health literacy, or significant cognitive impairment may have been underrepresented. This may have introduced selection bias and could limit the generalizability of the findings to these populations. To address these dimensions of user acceptance and patient-related constraints, future implementation studies should incorporate the Technology Acceptance Model (TAM). Although the patient-facing component of SiMON-PRO-Hematology® is currently limited to PROM and PREM completion, evaluating factors such as digital literacy, perceived ease of use, perceived usefulness, and security perceptions through the TAM framework will provide a robust methodology to understand, quantify, and mitigate these adoption barriers in both patients and healthcare professionals.^33^, ^34^, ^35^

Finally, future development should explore incorporating Artificial intelligence-driven predictive models using PROMs data,^36^, ^37^ while carefully addressing cybersecurity and ethical challenges^38^.

Additionally, future work will focus on rigorously evaluating the clinical impact of the system through prospective studies, which are crucial for quantifying its potential benefits in clinical outcomes and patient QoL.

In conclusion, SiMON-PRO-Hematology is a feasible and scalable tool designed to potentially enhance the quality and efficiency of pharmaceutical care in patients with hematological malignancies. Its design, based on structured data collection, the integration of PROMs, and real-time alerts, offers an innovative approach for monitoring treatment outcomes and patient well-being in daily practice. To fully objectify the benefit in care that this tool provides, it will be necessary to conduct prospective studies.

## CRediT authorship contribution statement

**Alejandro Martínez Pradeda:** Writing – original draft, Software, Resources, Methodology, Investigation, Data curation, Conceptualization. **Elena Fernández Gabriel:** Writing – review & editing, Supervision, Software, Resources, Project administration. **Sergio Jimeno Aguado:** Investigation, Formal analysis, Data curation. **Ana Sanclaudio Luhia:** Visualization, Software, Conceptualization. **Víctor Noriega Concepción:** Writing – review & editing, Supervision, Conceptualization. **Luis Margusino-Framiñán:** Writing – review & editing, Visualization, Validation, Project administration, Formal analysis, Conceptualization.

## Ethical considerations

As no primary data will be collected for the purposes of this study, no formal ethical approval is required.

## Funding

This research received no specific grant from any funding agency in the public, commercial, or not-for-profit sectors.

## Declaration of competing interest

The authors declare the following financial interests/personal relationships which may be considered as potential competing interests:

Alejandro Martinez Pradeda reports was provided by Complexo Hospitalario Universitario de A Coruña. If there are other authors, they declare that they have no known competing financial interests or personal relationships that could have appeared to influence the work reported in this paper.

## Data Availability

Data sharing not applicable to this article as no datasets were generated or analyzed during the current study.
