# Supplementary material for: SiMON-PRO-Hematology®: Digital techology for pharmacotherapeutic and patient reported outcomes monitoring in patients with hematologic malignancies
Source: Explor Res Clin Soc Pharm. 2026 Jun 26;23:100819. doi: 10.1016/j.rcsop.2026.100819 (PMC13330682; doi:10.1016/j.rcsop.2026.100819)
Supplement: Supplementary file 1 [file mmc1.zip › mmc1.odt]

ANNEX I: CHRONIC LYMPHOCYTIC LEUKEMIA (CLL) DATASET VARIABLES

1. PATIENT REGISTRATION

A) Educational Level

B) Baseline Comorbidities (All as "Yes / No" selections)

- Solid Tumor: Yes / No

- Hematologic Tumor: Yes / No

- Neurological Comorbidities (includes hearing loss): Yes / No

- Cognitive Impairment: Yes / No

- Urological Comorbidities: Yes / No

- Rheumatological and/or Inflammatory Comorbidities: Yes / No

- Psychiatric Comorbidities: Yes / No

- Endocrine Comorbidities (Excluding Hypothyroidism): Yes / No

- Respiratory Comorbidities (Excluding COPD): Yes / No

- COPD (Chronic Obstructive Pulmonary Disease): Yes / No

- Digestive Comorbidities: Yes / No

- Hypogammaglobulinemia: Yes / No

- CKD (Chronic Kidney Disease): Yes / No

- Hyperuricemia: Yes / No

- T2DM (Type 2 Diabetes Mellitus): Yes / No

- Prior Hypertension (HTN): Yes / No

- Dyslipidemia: Yes / No

- Obesity: Yes / No

- Fatty Liver: Yes / No

- Hypothyroidism: Yes / No

- ACS (Acute Coronary Syndrome): Yes / No

- Prior Stroke: Yes / No

- Prior Atrial Fibrillation (AF): Yes / No

- Aneurysm: Yes / No

- Heart Failure: Yes / No

C) Habits

- Alcohol Consumption: Never / Occasional / Previously / Daily

- Smoking Status: Non-smoker / Ex-smoker / Occasional / Daily / Not documented

2. TREATMENT LINES

(Variables introduced for each distinct treatment line)

A) Treatment Type in this Line

- Rituximab-Bendamustine

- Rituximab-Fludarabine-Cyclophosphamide (RFC)

- Rituximab-Chlorambucil

- Chlorambucil

- Ibrutinib

- Venetoclax

- Venetoclax-Rituximab

- Venetoclax-Obinutuzumab

- Idelalisib (Assumed translation for 'Idealmente')

- Acalabrutinib

- Zanabrutinib

- R-CHOP

B) Start Date

- $$Date Picker$$

C) Time Calculation

- Calculate Time from Previous Line's Last Dose.

D) Administration Setting at Start

- Inpatient / Outpatient

E) Suspension Date (Suspension)

- (Date Picker)

F) Reason for Suspension (Suspension)

- (Dropdown/Open Text)

G) Mutational Status

(Note: Selection of the last option should clear previous selections by default.)

  - Unknown

  - del(13q)

  - del(17p)

  - del(11q)

  - Trisomy 12

  - Unmutated IgHV

  - Complex Karyotype

  - Not Detected

  - Mutated IgHV

  - TP53 Mutation

  - Deletion 13S319

  - del(6q)

  - t(13;20) Translocation

  - Not Detected

H) New Comorbidities

- $$Open Text/List Selection$$

I) Transfusion Dependence at Line Start

- Yes / No

J) Antiplatelet Treatment

- Yes / No

  - J.1) Antiplatelet Treatment Start Date

  - J.2) Antiplatelet Treatment Suspension Date

K) Anticoagulant Treatment

- Yes / No

  - K.1) Anticoagulant Treatment Start Date

  - K.2) Anticoagulant Treatment Suspension Date

L) RAI Stage

- 0 / I / II / III / IV

M) BINET Stage

- A / B / C / Unknown

N) Number of Concomitant Treatments

- Numerical Value

Ñ) Concomitant Phytotherapy

- Yes/No or Open Text

O) Interactions

- Minor / Moderate / Contraindicated / None

P) Number of Interactions

- Numerical Value

Note on PROMs: It was agreed that PROMs (Patient-Reported Outcomes Measures) questionnaires must restart (count "from 0") with each new treatment line.

3. FOLLOW-UPS

(Two types: General Follow-up and Follow-up with Medication)

0) Consultation Type

- Telephone / In-person (or Face-to-face)

00) Attended By

- Patient / Relative (Family member) / Patient accompanied

A) Dose

- Enter value in milligrams (mg)

B) ADR (Adverse Drug Reaction) / Side Effect

- Yes / No

C) Ramp-up Phase

- Yes / No

D) Transfusion

- Yes / No

E) Adherence

- (Calculation based on fields for number of dispensed tablets, remaining tablets, and calculated adherence, similar to the SiMON-HCV model.)

E) Time Since Last Follow-up

- (Calculated Field)

F) Invasive Procedures

- Yes / No

G) Suspension due to Toxicity

- Yes / No

H) Dose Change due to Toxicity

- Yes / No

I) Lymph nodes

- No changes

- Radiological reduction / Visual reduction

- Radiological disappearance / Visual disappearance

- Radiological progression / Visual progression

4. EVENTS
