# Supplementary material for: SiMON-PRO-Hematology®: Digital techology for pharmacotherapeutic and patient reported outcomes monitoring in patients with hematologic malignancies
Source: Explor Res Clin Soc Pharm. 2026 Jun 26;23:100819. doi: 10.1016/j.rcsop.2026.100819 (PMC13330682; doi:10.1016/j.rcsop.2026.100819)
Supplement: Supplementary file 2 [file mmc2.zip › mmc2.odt]

SUPPLEMENTARY MATERIAL II: Cybersecurity, Network Architecture, and Data Protection Framework

To ensure the confidentiality, integrity, and availability of the sensitive medical data managed by the SiMON-PRO-Hematology® system, a multi-layered security architecture has been implemented. This infrastructure, fully managed by the institutional ICT Infrastructure Service (under the Sub-directorate of Information Systems and Technologies), specifically mitigates the security risks associated with deploying mobile tablets in hospital environments through three fundamental operational axes:

1. Device Security and Mobile Device Management (MDM) Institutional tablets utilized for patient-reported data collection operate under a centralized security model governed by a corporate Mobile Device Management (MDM) platform. Prior enrollment of each device automatically enforces strict hospital security policies and restricts the operating environment to the medical questionnaire interface via a single-app "Kiosk Mode". This completely prevents patients from accessing the underlying operating system, modifying system configurations, or installing unauthorized third-party software. Furthermore, the system adopts a strict zero-persistence policy; the tablet functions exclusively as a thin client, meaning that sensitive clinical data are transmitted in real time to secure servers and are never stored locally in the physical memory of the mobile device.

2. Network and Application Security Wireless connectivity is secured by restricting the tablets' access exclusively to a private, dedicated corporate Wi-Fi network that is continuously monitored by the organization's technological infrastructure. Confidentiality and data integrity in transit are guaranteed through Advanced Encryption Standard (AES with CCMP128) at the wireless link layer, coupled with WPA3/WPA2 Enterprise (802.1X) authentication. Data exchange via web/API calls is fully protected using TLS 1.2 and TLS 1.3 protocols, establishing a highly secure environment for hospital data capture. At the application level, SiMON-PRO-Hematology® incorporates a strict Role-Based Access Control (RBAC) framework integrated with corporate LDAP authentication, ensuring user access is restricted according to professional profiles and institutional permissions.

3. Reference Frameworks, Audit Trails, and Compliance Every interaction within the platform generates immutable, automated audit trails to ensure full traceability of data management under continuous ICT infrastructure monitoring. This entire technological environment and its operational guidelines are governed by the Sub-directorate of Information Systems and Technologies, whose management is formally certified under the National Security Scheme (ENS) and the international ISO/IEC 27001 standard. This compliance ensures complete alignment with the General Data Protection Regulation (GDPR) for the processing of high-level sensitive health data.
